# Dissociation between video head impulse test and caloric test: a marker of menière's disease? – A systematic review and meta-analysis

**DOI:** 10.1016/j.bjorl.2023.101279

**Published:** 2023-06-03

**Authors:** Jonas Belchior Tamanini, Raquel Mezzalira, Maria Gabriela Bonilha Vallim, Guilherme Paiva Gabriel, Guita Stoler, Carlos Takahiro Chone

**Affiliations:** Universidade Estadual de Campinas (UNICAMP), Departamento de Otorrinolaringologia, Campinas, SP, Brazil

**Keywords:** Meniere’s disease, Caloric test, Video head impulse test, Vestibulo-ocular reflex

## Abstract

**Objective:**

To analyze, by means of a systematic review and meta-analysis, the proportion of patients with Meniere’s disease who have altered caloric test and vHIT, as well as to determine the prevalence of altered caloric test and normal vHIT dissociation in the diagnosis of Meniere’s disease.

**Methods:**

The literature search had no restriction regarding the period of publication on the following indexed data platforms: PubMed, PubMed PMC, BVS-Bireme, Web of Science, Embase and Cochrane Library. Articles that evaluated patients with Meniere’s disease who underwent caloric test and vHIT were included. Two researchers independently conducted the analysis of the articles, promoting the selection and capture of data, following the recommendations of the PRISMA method, and complying with the criteria for articles inclusion and exclusion defined in the research protocol. In case of disagreement during the selection process, a third researcher was included for analysis.

**Results:**

From a total of 427 initial studies, the researchers selected 12 articles, published between 2014 and 2021, with a total of 708 patients evaluated, with a mean age of 52.72 years old. The prevalence of patients with Meniere’s disease with altered caloric reflex test was 64% (95% CI 57%‒71%), while the prevalence of altered vHIT was only 28% (95% CI 16%–40%). The prevalence of the altered caloric test + normal vHIT dissociation was 47% (95% CI 37%–57%).

**Conclusion:**

The video head impulse test and the caloric test are valuable tools for vestibular assessment. The dissociation of findings between these two tests in patients with Meniere’s disease was more prevalent in this meta-analysis and may be a result of the tonotopy of specialized hair cells in the ampullary crest. The prevalence of altered caloric test was 64% and anormal vHIT was 28%. The dissociation caloric asymmetry and normal vHIT was observed in 47% of the patients.

**Level of evidence:**

1.

## Highlights

• The prevalence of the altered caloric test + normal vHIT dissociation was 47%.
• The dissociation of findings between the two tests may be a result of the tonotopy of hair cells in the ampullary crest.
• This condition could help for the diagnosis of Meniere’s disease.

## Introduction

To date, Menière's disease is still considered a diagnostic and therapeutic challenge. According to the Bárány Society, this condition is characterized by two or more episodes of spontaneous vertigo, lasting between 20 min and 12 h, sensorineural hearing loss at low and medium frequencies in the affected ear and fluctuating otologic symptoms (hearing loss, tinnitus, and aural fullness) in the affected ear.^1^

Although the diagnosis of Menière's disease is essentially clinical, assessment of auditory and vestibular functions is fundamental in establishing differential diagnoses, as well as in patients’ treatment and follow-up.^2^

Among the tests that assess the Vestibulo-Ocular Reflex (VOR), the caloric test and the Video Head Impulse Test (vHIT) are the most used. The caloric test is the most accepted test for the study of peripheral vestibular function. However, it is a limited test, as it only stimulates the lateral semicircular canals at low frequencies.^2^, ^3^, ^4^

On the other hand, the vHIT is a fast-performing test that evaluates the six semicircular canals at high stimulus frequencies. It is more sensitive to the detection of saccades, especially “covert saccades”, and allows greater reliability in the VOR measurement, as well as its record.^3^, ^5^

Some studies reported a dissociation in the findings of these two tests in patients with Menière's disease, who had altered caloric test responses associated with a normal Vhit.^6^, ^7^

A possible explanation for the discrepancy between the results of the two tests is based on the different pathways of VOR stimulation of both or a consequence of the physical widening of the membranous duct in the hydropic labyrinth in Meniere’s disease.^7^ However, the question is whether the dissociation of such results can suggest the diagnosis of Menière's disease.

The objective of this study is to analyze, through systematic review and meta-analysis, the proportion of patients with Meniere's disease who have altered caloric test and vHIT, as well as to determine the prevalence of altered caloric test and normal vHIT dissociation in the diagnosis of Meniere's disease.

## Methods

### Search strategy and data sources

This study used the recommendations of the Preferred Reporting Items for Systematic Review and Meta-Analyses (PRISMA) method.^8^ The literature search had no restriction regarding the period of publication on the following indexed data platforms: PubMed, PubMed PMC, BVS-Bireme, Web of Science, Embase and Cochrane Library. Gray literature was consulted through the Brazilian Digital Library of Theses and Dissertations (BDTD) and EBSCOHOST. The following descriptors were used as a search strategy in this research: “Menière's disease” AND “Caloric vestibular test” OR “Video-head impulse test (v-HIT)” OR “Video Head Impulse test (VHIT)”.

### Eligibility criteria for studies selection

The studies selected for this meta-analysis were established using the PICO strategy (Patient, Intervention, Comparison and Outcome), with individuals with Meniere’s Disease aged 18 or older as target population. It was considered as an intervention the performance of tests that evaluate the vestibular function in this condition – vHIT and Caloric Test ‒ as well as the comparison between these two tests. Thus, the studies eligible for this research should present a consistent and comparative analysis between these two tests in patients diagnosed with Meniere’s disease.

The following were considered as exclusion criteria: (1) Inadequate target population (under 18 years old); (2) Experimental studies *in vitro*; (3) Inappropriate study types: simple reviews, abstracts, letters to the editor, case reports; (4) Insufficient clinical information; (5) Language that does not include Portuguese, English or Spanish; (6) Improper outcome.

**Figure 1:**
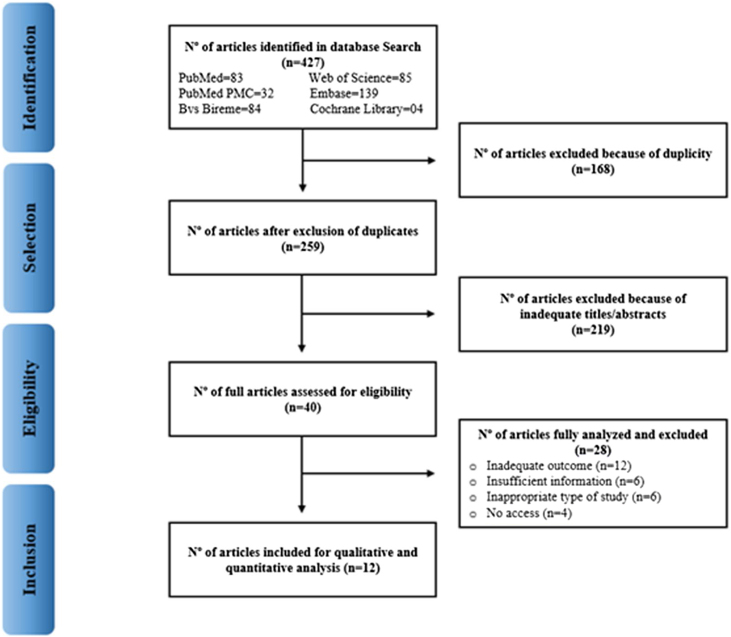
Flowchart of article selection.

Initially, the titles and abstracts of all articles were read independently by two researchers. After that, according to the eligibility criteria, the pre-selected articles were read in full for the composition of the meta-analysis. In case of disagreement during the selection, a third researcher performed analysis. The selection of studies is shown in Fig. 1.

### Data extraction

Data extraction was performed in a standardized way, consisting of year of publication, authors, sample size, mean age of patients included, study design, in addition to the proportion of patients with Menière's disease who had abnormal vHIT and caloric test, as well as information about the combination of findings from these two tests, when present in the articles.

### Group selection

After data extraction, the study consisted of two stages. First, the objective was to evaluate the prevalence of patients with Menière's disease who presented changes in caloric test and vHIT alone. In a second moment, the objective was to evaluate the prevalence of the combination of results of these two tests to analyze the combination through four groups: (1) Normal caloric test and vHIT; (2) Altered caloric test and normal vHIT; (3) Altered caloric test and vHIT; (4) Normal caloric test and altered vHIT.

### Methodological quality assessment

The Agency for Health Care Research and Quality (AHRQ) checklist was used to assess methodological quality and publication bias. It uses 11 criteria to evaluate the studies included in research, namely source of information, inclusion and exclusion criteria, period of time, consecutive patients, masking, quality assurance, explanation of exclusions, control of confounding factors, withdrawal of incomplete data, complete data collection and follow-up of research patients. In each of the criteria, the score is 1 if present in the study or 0 if not present. Thus, a high methodological quality refers to articles with a score equal to or greater than 8 (Table 1).^9^

**Table 1:** Quality control of the selected studies according to the Agency for Health Care Research and Quality (AHRQ) criteria.

	Methodological quality of the article (AHRQ)											
Articles	A	B	C	D	E	F	G	H	I	J	K	Score
Blodow A et al.^10^	1	1	1	1	0	1	1	1	0	1	0	8
Cordero-Yanza J et al.^2^	1	1	1	1	0	1	1	1	0	1	0	8
Eza Nunez P et al.^11^	1	1	0	1	0	1	1	1	1	1	0	8
Fukushima M et al.^12^	1	1	1	1	0	1	0	1	1	1	1	9
Hannigan IP et al.^13^	1	1	1	1	0	1	0	1	1	1	0	8
Kitano K et al.^14^	1	1	1	1	0	1	0	1	1	1	0	8
Limviriyakul et al.^15^	1	1	1	1	0	1	1	1	0	1	0	8
Oliveira LNR et al.^16^	1	1	1	1	0	1	1	1	0	1	0	8
Rubin F et al.^17^	1	1	1	1	0	1	1	1	0	1	0	8
Sobby OA et al.^18^	1	1	0	1	0	1	1	1	1	1	0	8
van Esch BF et al.^19^	1	1	1	1	0	1	0	1	1	1	0	8
Zhou R et al.^20^	1	1	1	1	0	1	1	1	1	1	0	9

A, Information Source; B, Inclusion or exclusion criteria; C, Period of time; D, Consecutive patients; E, Masking; F, Quality assurance; G, Exclusion explanation, H, Confusion factors control; I, Incomplete data removal; J, Data integrity; K, Patient follow-up; 1, Present information; 0, Absent or uncertsin information.

### Statistical analysis

The R software (R Version 4.1.0. Copyright© 2021 The R Foundation for Statistical Computing) was used for statistical analysis of this meta-analysis and random models using the Restricted Maximum Likelihood Method (REML) were used to estimate the prevalence.

The assessment of heterogeneity between studies was performed using the *Q* test, and the *I*² statistic was used for the quantitative analysis of heterogeneity. This method estimates the proportion of heterogeneity observed in the studies, which can vary from 0% to 100%. The higher the value, the greater the differences between the studies.

Checking for the presence of outlier was performed through externally studentized residual. The leave-one-out method was used to detect influential studies. To compare the studies, a random model was adjusted for each analysis and a fixed model to combine them. The heterogeneity within each group was assumed the same and the Wald test was used to compare the prevalence of the combination of results from these two tests, which enabled to analyze the combination through 4 groups: (1) Normal caloric test and vHIT; (2) Altered caloric test and normal vHIT; (3) Altered caloric test and vHIT; (4) Normal caloric test and altered vHIT. The level of significance adopted was 5% (*p* < 0.05).

## Results

A total of 12 articles were included, published between 2014 and 2021, with 708 patients evaluated, with a mean age of 52.72 years old. All studies specifically evaluated patients with Menière's disease, according to the diagnostic criteria of the American Academy of Otolaryngology-Head and Neck Surgery, 1995, and Bárány Society, 2015, who underwent both caloric test and vHIT (Table 2). In ten included studies, it was possible to analyze the results in four groups based on the findings of both tests: (1) Normal caloric test and vHIT; (2) Altered caloric test and normal vHIT; (3) Altered caloric test and vHIT; (4) Normal caloric test and altered vHIT (Table 3). The parameters used to evaluate the caloric reflex test and vHIT are described in Table 4.

**Table 2:** Characteristics evaluated in the selected studies.

Author	Year	Study	Patients M/F	Age (mean)	Altered Vhit	Altered caloric test	Criteria
Blodow A et al.	2014	Transversal	30 (9/21)	54	11 (36.6%)	20 (66.6%)	AAO-HNS 1995
Cordero-Yanza J et al.	2017	Retrospective	88 (45/43)	55	58 (65.9%)	59 (67%)	AAO-HNS 1995
Eza Nunez P et al.	2019	Transversal	50 (23/27)	55.5	11 (22%)	33 (66%)	AAO-HNS 1995
Fukushima M et al.	2019	Prospective	90 (30/60)	56.6	51 (56.7%)	37 (41.1%)	AAO-HNS 1995
Hannigan IP et al.	2021	Retrospective	73^a^	57	21 (28.7%)	48 (65.7%)	Bárány Society 2015
Kitano K et al.	2019	Retrospective	20 (9/11)^b^	50.9	2 (8%)	16 (64%)	Bárány Society 2015
Limviriyakul et al.	2020	Transversal	51 (13/38)	54.9	24 (47.1%)	39 (76.5%)	AAO-HNS 1995
Oliveira LNR et al.	2019	Transversal	32 (10/22)	45.7	10 (31.2%)	22 (98.7%)	Bárány Society 2015
Rubin F et al.	2018	Prospective	37 (13/24)	56	0 (0%)	31 (83.8%)	Bárány Society 2015
Sobby OA et al.	2019	Case control	40 (25/15)	43.3	8 (20%)	14 (35%)	AAO-HNS 1995
van Esch BF et al.	2018	Retrospective	89 (42/47)	55	21 (23.5)	64 (71.9%)	AAO-HNS 1995
Zhou R et al.	2020	Retrospective	98 (50/48)	49.6	6 (6.1%)	63 (64.3%)	AAO-HNS 1995

M, Male; F, Female; AAO-HNS, American Academy of Otolaryngology-Head and Neck Surgery.

^c^Only 82 of the 90 patients underwent caloric test.

a No information about sex in the study.

b From the 20 patients evaluated, 5 had bilateral Meniere’s disease.

**Table 3:** Comparison between caloric reflex test and vHIT findings.

Author	Normal caloric test + Normal vHIT	Altered caloric test + Normal vHIT	Altered caloric test + Altered vHIT	Normal caloric test + Altered vHIT
Cordero-Yanza J et al.	15.9%	18.2%	48.9%	17%
Eza Nunez P et al.	34%	44%	22%	0%
Fukushima M et al.	25.6%	18.2%	26.8%	29.2%
Hannigan IP et al.	34.2%	36.9%	28.7%	0%
Kitano K et al.	36%	56%	8%	0%
Limviriyakul et al.	7.8%	35.2%	41.1%	15.6%
Oliveira LNR et al.	43.6%	43.6%	12.8%	0%
Rubin F et al.	16.2%	94%	0%	0%
van Esch BF et al.	14.6%	74.1%	9.5%	1.6%
Zhou R et al.	38.8%	58.2%	6.1%	0%

**Table 4:** Parameter used for performing vHIT and caloric reflex test in the studies analyzed.

		Video Head Impulse Test (vHIT)		Caloric test	
Author	Year	Stimulus used	Altered criteria	Stimulus used	Alteration criteria
Blodow A et al.	2014	>10 head impulses, A 15–20 °, D 150–200 ms, VP 200 °/s	Gain <0.79, saccades and AR > 8.5%	Water (30 °/44 °C)	UW > 25%
Cordero-Yanza J et al.	2017	20 head impulses, PV 150–175 °/s	Gain < 0.8 (horizontal canals) or <0.7 (vertical cannals) and saccades	Water (30 °/44 °C)	UW > 20% and DP ≥ 28%
Eza Nunez P et al.	2019	20 head impulses, A 10‒20°	Gain <0.8 and saccades	Water (warm/cold)	UW > 25%
Fukushima M et al.	2019	>20 head impulses, PV 100–250 °/s	Gain <0.8 (horizontal canals) or <0.7 (vertical canals) and saccades	Water (30 °/44 °C)	UW > 25%
Hannigan IP et al.	2021	>20 head impulses, PV 100–300 °/s	Gain <0.8 and saccades	Water (30 °/44 °C)	UW ≥ 30%
Kitano K et al.	2019	>20 head impulses, PV 100–250 °/s	Gain < 0.8 and saccades	Water (20 °/44 °C)	AVSP ≤ 10 °/s
Limviriyakul et al.	2020	>20 head impulses, A 10–20 °, PV 150–200 °/s	Gain <0.8 (horizontal canals) or <0.7 (vertical cannals) and saccades	Air (24 °/50 °C)	UW > 25%
Oliveira LNR et al.	2019	>20 head impulses, A 15–20 °, PV 80–250 °/s	Gain <0.8 (horizontal canals) or <0.75 (vertical cannals) and saccades	Air (24 °/50 °C)	UW ≥ 20% and AVSP < 5°/s
Rubin F et al.	2018	>5 head impulses, A 10–20 °, PV > 120 °/s	Gain <0.78 (horizontal canals) or <0.64 (vertical cannals) and saccades	Water (30 °/44 °C)	UW > 20%
Sobby OA et al.	2019	5 head impulses, A 15‒20°	Gain <0.8 and saccades	Water (30 °/44 °C)	UW ≥ 25%
van Esch BF et al.	2018	>20 head impulses, A 10–20 °, D 150–200 ms, PV > 150 °/s	Gain <0.8 and saccades	Water (warm/cold)	UW ≥ 22% and DP ≥ 28%
Zhou R et al.	2020	>20 head impulses, A 5–15 °, PV 150–250 °/s	Gain <0.8 and saccades	Air (24 °/50 °C)	UW ≥ 25

A, Amplitude; D, Duration; PV, Peak velocity; AR, Asimmetry ratio; UW, Unilateral weakness; DP, Directional Preponderance; AVSP, Angular velocity of the slow phase.

### Comparison between caloric test and vHIT

**Figure 2:**
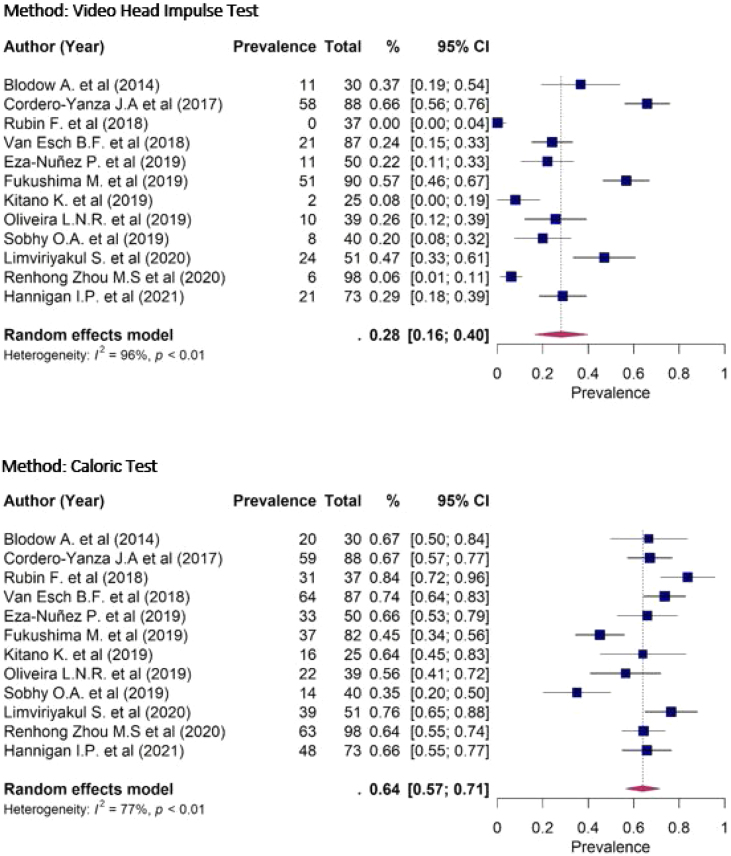
Forest plot of the prevalence of alterations in vHIT and caloric test.

A comparison was made between the prevalence of alterations in the caloric test and vHIT in the 12 included studies, with 700 patients evaluated with the caloric test and 708 patients evaluated with vHIT. The prevalence of patients with altered caloric test was 64% (95% CI 57%–71%), with high heterogeneity between the articles, evidenced by both the *Q* test (*p* < 0.01) and the *I*^2^ measure (77%). On the other hand, the proportion of patients with altered vHIT was only 28% (95% CI 16%–40%) and, similarly, also showed high heterogeneity between the articles, through the *Q* test (*p* < 0.01) and *I*^2^ measurement (96%) (Fig. 2).

### Dissociation between caloric reflex test and vHIT

In a second meta-analysis, the prevalence of the combination of findings between caloric reflex test and vHIT was evaluated, making it possible to subdivide it into 4 groups: (1) Normal caloric test and vHIT; (2) Altered caloric test and normal vHIT; (3) Altered caloric test and vHIT; (4) Normal caloric test and altered vHIT. At this stage, only ten articles were included, since the others did not have enough data to allocate patients to the aforementioned groups.

**Figure 3:**
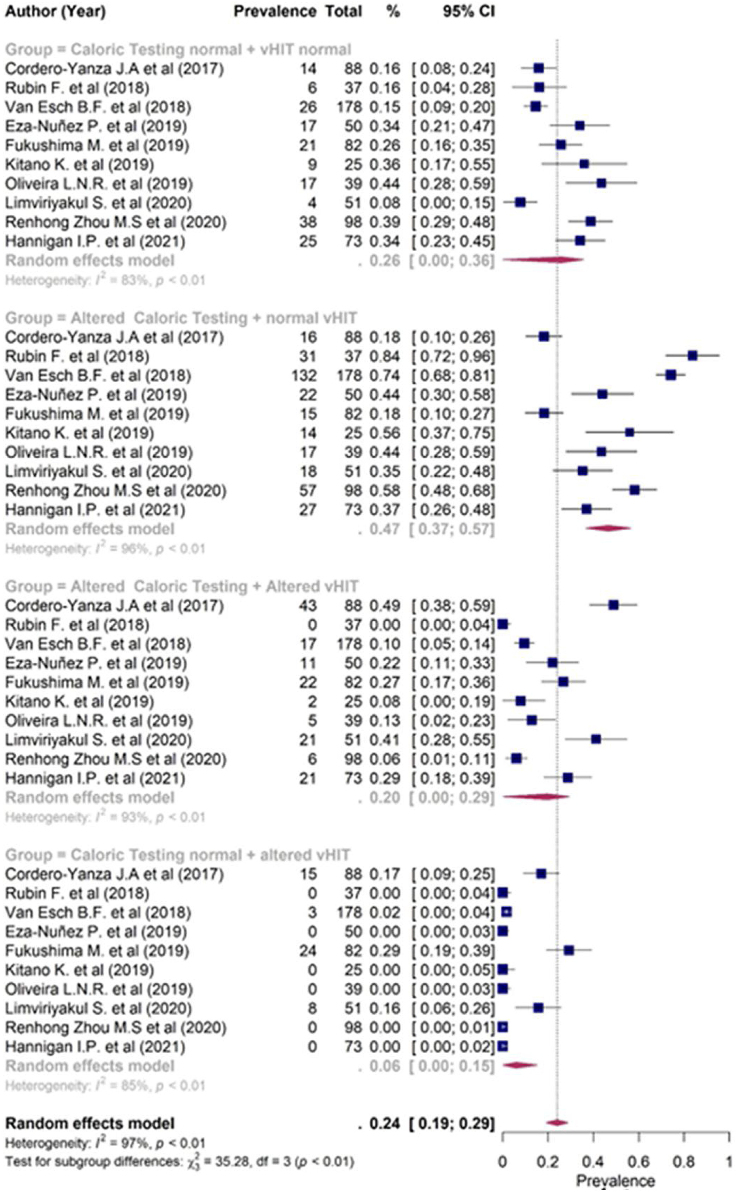
Forest plot of the prevalence of alterations between the combining groups and findings between caloric test and vHIT.

The prevalence analysis of both normal caloric test and normal vHIT (Group 1) was 26% (95% CI 0%–36%), while the prevalence of the association of altered caloric test and normal vHIT (Group 2) was 47% (95% CI 37%–57%). Evaluation of both altered exams (Group 3) resulted in a prevalence of 20% (95% CI 0%–2%) and the prevalence of patients with only vHIT altered (Group 4) was 6% (95% CI 0%–15%) (Fig. 3). A statistically significant difference was observed in patients with altered caloric test and normal vHIT (Group 2) in relation to the other groups (*p* < 0.001), and the other groups did not show significant differences between them.

## Discussion

Several studies have previously suggested the dissociation of caloric test and vHIT as a marker of Menière's disease, however, the lack of literature so far prevented the confirmation of this affirmation. Thus, our objective was to analyze, through a systematic review and meta-analysis, the proportion of patients with Menière's disease who have altered caloric test and vHIT, as well as to determine the prevalence of altered caloric reflex test and normal vHIT dissociation in the patients of Menière's disease.

Nine^2^, ^10^, ^11^, ^12^, ^13^, ^14^, ^17^, ^18^, ^19^ of the 12 articles analyzed showed caloric tests were performed with water (6 of the them at temperatures of 30 °C and 44 °C),^2^, ^10^, ^11^, ^12^, ^13^, ^17^, ^18^ while in three of the studies air (at temperatures of 24 °C and 50 °C)^15^, ^16^, ^20^ was used as a stimulus to perform the same test, which is equivalent when performed at these temperatures.^21^ In 11 articles, labyrinthine predominance was used as a criterion for altering the caloric test, which varied between the values of ≥22% and ≥30%. In only one study, the value of the angular velocity of the slow phase ≤10º/s was considered as the only change criterion.^14^ The AVSP can be used to analyse the caloric tests, as the unilateral weakness. For this reason, the article was included in our study. In two other studies, directional preponderance was also considered, and values ≥28% were classified as altered.^2^, ^19^ On the other hand, in relation to vHIT, all tests were performed in a standardized way, with short stimuli, amplitude between 5 ° and 20 ° and with peak velocity in most studies varying between 80% and 300 °/s. In all articles, the change in the VOR gain was considered as a criterion for alteration the test. In ten studies, the gain value considered altered was < 0.8 in horizontal semicircular canals, while one study considered <0.79^10^ and another considered a value of <0.78.^17^ All studies included the presence of overt and covert saccades as a change in the test, as well as in one of the articles the asymmetry ratio between the ears was calculated.

Five articles^2^, ^12^, ^15^, ^16^, ^17^ evaluated the 6 semicircular canals in the vHIT. Two of them^2^, ^12^ took into account the results of all canals in the comparison with the caloric test and the others only used data from the horizontal canal or did not make it clear which data were compared. The combination of altered caloric test and normal vHIT in the articles by Cordero-Yanza et al. and Fukushima et al. was 18.2%. However, when we consider only the horizontal semicircular canal we can observe that the combination of altered caloric test and normal vHIT is 45.5% and 34.1%, respectively. According to Cordero-Yanza et al., when only the horizontal semicircular canal is analyzed, the agreement between both tests is poor, but if the vHIT is classified as abnormal when any of the 6 canals is altered the agreement between both tests remains poor.^2^

When comparing the prevalence of alterations between the two tests, in 64% of the patients the caloric test was found to be altered, while the proportion of patients with altered vHIT was only 28%. On the other hand, the association of altered caloric test and normal vHIT was present in 47% of the patients, and this difference was statistically significant when compared to the other combinations of results from these tests.

The dissociation of results in the caloric reflex test and vHIT can be explained by the anatomy and physiology of the crista ampullaris, the main receptor of the VOR. This structure is composed of specialized type I and type II hair cells, which have specific distributions in the crista. Type I hair cells occupy mainly the central region of this structure and are responsible for decoding stimuli of head movement at high frequencies and acceleration. Irregular afferent fibers go from these cells toward the excitatory neurons, located in the vestibular nucleus and conduct the ampullaris stimulus directly to the effector eye muscles, which is evaluated through vHIT. On the other hand, type II hair cells are peripherally located in the crista ampullaris, acting on stimuli and movements of low frequency and acceleration. From them come regular afferent fibers that synapse in the vestibular nucleus with inhibitory neurons that act on the internuclear inhibitory commissural pathway, promoting inhibition of the contralateral vestibular nuclei on the stimulated side, this pathway being analyzed through the caloric test.^22^, ^23^

One hypothesis is that there may be a selective impairment of the different regions of the crista, justifying the discrepancy in the results of tests that operate at different frequencies. This dissociation is well documented in Menière's disease literature and was described by Tsuji et al., in 2000.^23^ A possible explanation for this fact is based on the crista anatomy itself. Type II cells, as they occupy the periphery of the crista, are potentially susceptible to the influence of toxic metabolites in the perilymph, such as the accumulation of potassium. On the other hand, type I cells, as they occupy the most central portion of the crista and are surrounded by the calyx of the nerve ending, are isolated from the surrounding environment, therefore, more protected. The selective loss of type II cells would justify the preservation of the VOR fast pathway evaluated by vHIT.

Another explanation for the discrepancy between caloric test and vHIT in Menière's disease was recently published by McGarvie et al. and considers the hypothesis that endolymphatic hydrops would promote an increase in the diameter of the semicircular canals.^7^ The most accepted theory to explain the thermal response is the formation of convection currents caused by temperature variation in the endolymph that stimulate the crista ampullaris. The cupula accompanies the endolymph in a monobloc, deflecting the cilia of the hair cells and changing their neural firings in relation to rest.^25^ The enlargement of the semicircular canals, as a result of endolymphatic hydrops, would allow endolymph recirculation during caloric stimulation, which would reduce the pressure gradient in the cupula and, consequently, reduce the deflection of specialized hair cells in the caloric reflex test.^7^ This change would not have repercussions on vHIT, since the responses depend on the endolymph movement generated by the head stimulus and would not be influenced by the dilation of the endolymphatic space.

Both the caloric test and the vHIT consist of validated methods for vestibular assessment through the analysis of the VOR, however, they consist of tests that are considered complementary to each other, as they assess the peripheral vestibular system at different frequencies. While the first exam is capable of evaluating at lower frequencies (0.003 Hz), the vHIT consists of high-frequency stimuli (between 4 and 7 Hz).

Thus, it is possible that Menière's disease preferentially causes impairment in the vestibular apparatus responsible for processing low-frequency responses, justifying the presence of alterations in the caloric test, to the detriment of a vHIT within the normal range.^24^, ^25^, ^26^

On the other hand, another plausible explanation for such findings would be related to the central adaptation mechanism to the damage induced by Meniere's disease, which would be effective only for high-frequency physiological stimuli, not being present in low-frequency non-physiological stimuli.^25^, ^27^

However, a limitation of the present study is the high heterogeneity among the articles included in this meta-analysis, evaluated both by the *Q* Test and by the *I*^2^ measure. Thus, it is necessary to develop new studies later with less heterogeneity and standardized methods for patient selection to corroborate the findings of this present research.

## Conclusion

In Meniere’s disease, the prevalence of altered caloric test was 64% and anormal vHIT was 28%. Caloric asymmetry in the presence of normal vHIT was observed in 47% of the patients.

## Conflicts of interest

The authors declare no conflicts of interest.
